# Community composition and physiological plasticity control microbial carbon storage across natural and experimental soil fertility gradients

**DOI:** 10.1038/s41396-023-01527-5

**Published:** 2023-10-18

**Authors:** Orpheus M. Butler, Stefano Manzoni, Charles R. Warren

**Affiliations:** 1 School of Life and Environmental Sciences, The University of Sydney, Sydney, NSW, Australia; 2 Stockholm University and Bolin Centre for Climate Research, Stockholm, Sweden

**Keywords:** Biogeochemistry, Microbial ecology, Physiology

## Abstract

Many microorganisms synthesise carbon (C)-rich compounds under resource deprivation. Such compounds likely serve as intracellular C-storage pools that sustain the activities of microorganisms growing on stoichiometrically imbalanced substrates, making them potentially vital to the function of ecosystems on infertile soils. We examined the dynamics and drivers of three putative C-storage compounds (neutral lipid fatty acids [NLFAs], polyhydroxybutyrate [PHB], and trehalose) across a natural gradient of soil fertility in eastern Australia. Together, NLFAs, PHB, and trehalose corresponded to 8.5–40% of microbial C and 0.06–0.6% of soil organic C. When scaled to “structural” microbial biomass (indexed by polar lipid fatty acids; PLFAs), NLFA and PHB allocation was 2–3-times greater in infertile soils derived from ironstone and sandstone than in comparatively fertile basalt- and shale-derived soils. PHB allocation was positively correlated with belowground biological phosphorus (P)-demand, while NLFA allocation was positively correlated with fungal PLFA : bacterial PLFA ratios. A complementary incubation revealed positive responses of respiration, storage, and fungal PLFAs to glucose, while bacterial PLFAs responded positively to PO_4_^3-^. By comparing these results to a model of microbial C-allocation, we reason that NLFA primarily served the “reserve” storage mode for C-limited taxa (i.e., fungi), while the variable portion of PHB likely served as “surplus” C-storage for P-limited bacteria. Thus, our findings reveal a convergence of community-level processes (i.e., changes in taxonomic composition that underpin reserve-mode storage dynamics) and intracellular mechanisms (e.g., physiological plasticity of surplus-mode storage) that drives strong, predictable community-level microbial C-storage dynamics across gradients of soil fertility and substrate stoichiometry.

## Introduction

The acquisition, storage, and release of carbon (C) and nutrients by soil microorganisms underpins the function of terrestrial ecosystems [1–3], and the efficiency of microbial C use is among the most important controls over soil organic carbon accumulation [4–6]. One factor that likely influences these essential biogeochemical processes is the ability of microorganisms to synthesize C-rich compounds that seemingly serve as intracellular pools of stored C [7, 8]. Such compounds include triacylglycerides (TAGs) and trehalose, which are common across eukaryotes and prokaryotes, and polyhydroxyalkanoates (e.g., polyhydroxybutyrate [PHB]), which are found only in prokaryotes (i.e., bacteria). These compounds, which can account for a large fraction of dry cell weight (e.g., 70–80 % in the case of PHB) [9–11], accumulate under suboptimal conditions and can enhance microbial survival in variable, unfavourable, or resource-deficient environments [12–15]. Moreover, stoichiometric models indicate that intracellular C-storage can mitigate stoichiometric imbalances between microbes and substrate, leading to improved microbial nutrient and C use efficiency (CUE) compared to storage-free scenarios [8]. Thus, intracellular C-storage is potentially important for the function of terrestrial ecosystems and – through its influence over microbial CUE – the net conversion of atmospheric CO_2_ to soil organic C [4–6]. However, our present understanding of microbial C-storage draws heavily on culturing studies [16–18], industrial research [9, 19], and marine ecosystems [20, 21]. Few studies have examined C-storage in natural communities of soil microorganisms [7], such that our understanding of the dynamics and functionality of soil microbial C-storage in terrestrial ecosystems is incomplete.

Modelling indicates that the benefits of intracellular C-storage for microbial nutrient and C use efficiency increase with substrate C:nutrient ratio [8]. This suggests that the extent and functional significance of intracellular C-storage could vary with soil fertility and reach their maxima in ecosystems associated with strongly weathered, nutrient-depleted soils. In such ecosystems, many biological processes are limited by phosphorus (P) [22–25], with sustained ecosystem function contingent on efficient microbial P cycling that gradually remobilises P for plant uptake while minimising ecosystem P losses via leaching [26]. However, most studies of soil microbial C-storage have focussed on fertile soils in temperate regions [27–32]. To our knowledge, no studies have explored the dynamics or function of microbial C-storage across natural gradients of soil fertility and substrate stoichiometry.

Moreover, in natural soils, microbial C-storage is typically quantified across the entire soil microbiome [27–33], such that intracellular drivers of C-storage dynamics are potentially superimposed over community-level drivers (e.g., changes in taxonomic composition). Intracellular drivers—i.e., physiological plasticity of individual cells—have received substantial attention in prior studies of microbial isolates ex situ [15, 16, 34]; by contrast, community-level drivers are largely unstudied. Thus, the relative contributions of intracellular and community-level processes to community-level microbial C-storage dynamics in natural soils are unknown. Disentangling these hierarchical and potentially interacting drivers of storage dynamics could enhance our ability to predict microbially mediated biogeochemical processes, including rates of nutrient mineralisation and soil C accumulation across environmental gradients and in response to environmental change.

**Fig. 1:**
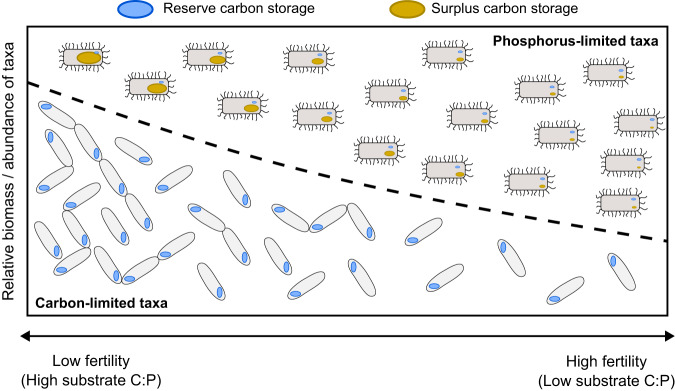
Conceptual depiction of hypothesised community-level soil microbial carbon (C) storage dynamics across natural and/or experimental gradients of soil fertility. Microbial taxa above the dashed line have high phosphorus (P) requirements and thus tend toward P-limited growth, while taxa below the dashed line have comparatively low requirements for P, but high requirements for C, due in part to their high constitutive allocation to reserve C-storage, such that these taxa tend toward C-limited growth. The biomass and/or abundance of P-limited taxa relative to C-limited taxa declines from high fertility to low fertility soils, but their intracellular allocation of C to surplus storage increases via physiological plasticity due to increasingly strong P-limitation associated with high substrate C:P ratios [64]. Conversely, the relative biomass/abundance of C-limited taxa increases relative to P-limited taxa from high fertility to low fertility soils, leading to an increase in community-level reserve C.

One potential framework for disentangling the intracellular and community-level drivers of soil microbial C-storage dynamics across soil fertility gradients is provided by the conceptual “surplus” and “reserve” storage modes (Fig. 1) [7, 35]. In principle, changes in surplus mode C-storage occur via physiological plasticity of individual cells in response to an over-supply of C relative to microbial requirements for growth and maintenance (i.e., nutrient limitation) [8]. By contrast, allocation of C to reserve storage is constitutive, occurring as a fixed, taxon-specific proportion of acquired C and incurring a trade-off against short-term growth [7, 8, 35]. In periods of extreme resource scarcity, reserve storage can be remobilised to support survival or growth [7, 35]. In theory, surplus C-storage has stronger positive effects on microbial nutrient and C use efficiency than reserve C-storage [7, 8]. In the case of CUE, this is because some fraction of excess substrate C can be flexibly allocated to surplus storage instead of lost through “overflow” respiration. High microbial CUE drives high rates of bulk soil organic C sequestration [4–6]; thus, distinguishing and delineating the drivers of the two modes will enhance our understanding of the dynamics and functional implications of soil microbial C storage in natural ecosystems.

Given the constitutive nature of reserve storage, it is reasonable that variation in microbial community-level reserve storage allocation (i.e., storage scaled to non-storage biomass) is driven primarily by community composition. By contrast, changes in surplus storage allocation do not necessarily entail compositional changes. Furthermore, the trade-off against growth associated with reserve storage means that a higher constitutive allocation to reserve C-storage by a given taxon increases that taxon’s tendency toward C-limited growth. Thus, given that C-limited taxa tend to dominate environments with high substrate C:P, whereas P-limited taxa tend to dominate environments with low substrate C:P [36, 37], we reason that declining soil fertility—and correspondingly high substrate C:P—should be associated with enhanced community-level microbial C-storage due to (1) a higher relative abundance of microorganisms that constitutively allocate large amounts of C to the reserve mode, and (2) a greater intracellular allocation of C to the surplus mode by P-limited microorganisms (Fig. 1). Given that fungi and bacteria tend toward nutrient- and C-limitation, respectively [38, 39], and that TAGs and PHB respectively tend to serve primarily the reserve and surplus modes (although both compounds potentially serve both modes [7, 32]), we further expected that surplus storage would be represented by PHB associated with P-limited bacteria, while reserve storage would be represented by TAGs associated with C-limited fungi.

To test these hypotheses, we quantified neutral lipid fatty acids (NLFAs—a proxy for TAGs), PHB, and trehalose across several soil types in eastern Australia. We also quantified microbial polar lipid fatty acids (PLFAs; i.e., membrane lipids [excluding glycolipids], which in P-deficient soils are comprised of both phospholipids and betaine lipids [40, 41]) and variables associated with soil fertility and microbial resource demand. We then tested the short-term responses of microbial growth, respiration, and C-storage to manipulation of substrate C:P in a 10-day incubation featuring two contrasting soils from our fertility gradient. Finally, we used these soils to parameterise an existing model of microbial C allocation [8], enabling us to compare our empirical results with model predictions derived from current knowledge of microbial growth and storage physiology.

## Materials and methods

### Study sites and sampling

**Fig. 2:**
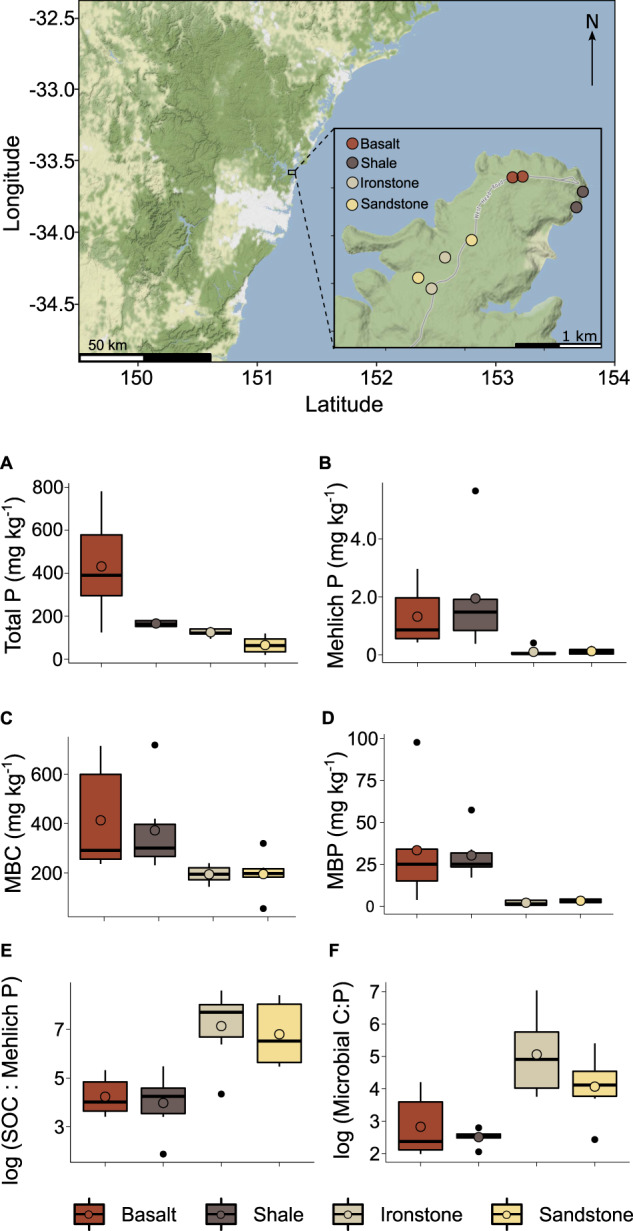
Natural gradient of soil fertility in eastern Australia. Map of the eight study sites established within the West Head section of Ku-Ring-Gai Chase National Park, New South Wales, Australia, across four soil types derived from either basalt, shale, ironstone, or sandstone parent material, and surface soil (0–10 cm) contents of (**A**) total phosphorus (P), (**B**) Mehlich-extractable (i.e., “available”) PO_4_^3—^P, (**C**) microbial biomass carbon (MBC), (**D**) microbial biomass P (MBP) contrasted among the four soil types, along with the ratios of (**E**) soil soluble (i.e., 0.5 M K_2_SO_4_-extractable) organic carbon (SOC) to Mehlich-extractable PO_4_^3—^P and (**F**) MBC to. There were six observations per soil type. Ratios were calculated on a mass basis (i.e., g C per g P) prior to natural log transformation.

Our study leveraged a natural gradient of soil fertility within a ~3.1 km^2^ area of the West Head section of the Ku-Ring-Gai Chase National Park (hereafter “West Head”) in eastern Australia (-33.59, 151.29; Fig. 2). This gradient is underpinned by spatial variation in parent material, with four surface lithologies present: middle Triassic sandstone and ironstone, early Triassic shales, and a basalt diatreme of early Tertiary origin [42]. For each soil type, we established six sampling points spread across two distinct locations (Fig. 2). At each location, there were three sampling points within a 40 m^2^ area, with all sampling points separated by >10 m. At each sampling point, five soil cores (0–10 cm depth) were collected after removal of plant litter. These cores were combined to give one composite soil sample per sampling point. Sampling was carried out on a single day in February 2022. Sampling points were treated as replicates in our analyses (n = 6 per soil type).

### Laboratory analyses of soil properties

Basic soil properties were measured following standard protocols (see Supplementary Information). Microbial biomass C (MBC) was measured via chloroform-fumigation vacuum-infiltration using the correction factor of 2.64 [43]. Extractions of storage compounds were carried out within 24 hours of sampling ( <12 hours for NLFA/PLFA and PHB) on unsieved soil sub-samples. For quantification of NLFAs and PLFAs, lipids were extracted quantitatively using the Bligh and Dyer method with a citrate aqueous phase (0.15 M, pH 4.0) [44]. Extracts (in a CHCl_3_ matrix) were subjected to silica SPE fractionation (Discovery DSC-Si SPE Tube bed wt. 100 mg, volume 1 mL). Eluates were dried under a stream of N_2_ gas before preparation for gas chromatography-mass spectrometry (GC-MS) via mild alkaline methanolysis (incubation at 37 °C for 30 minutes with 0.2 M methanolic KOH and CHCl_3_). Purified, methanolysed samples were dried under N_2_ gas and then re-dissolved in hexane with 25 µg ml^-1^ methyl heptadecanoate-d_33_ (Sigma-Aldrich) as an internal standard and analysed by GC-MS (GCMS-QP2010Plus, Shimadzu, Kyoto, Japan). The GC-MS was fitted with an arylene-modified 5% diphenyl– 95% dimethyl polysiloxane stationary phase (30 m long × 0.25 mm internal diameter × 0.25 mm film thickness; Rxi-5SilMS, Restek, Bellfonte, USA) and used helium as a carrier gas. FAMES in our samples were identified and quantified based on standards from commercial FAMES mixtures (a fatty acid methyl ester mix with 37 FAMES and a bacterial fatty acid methyl ester mix with 27 FAMES), retention indices based on even-numbered alkanes from C8 through to C36, and EI mass spectra from the NIST library. Microbial taxonomic assignments for FAMES followed a previous publication [45].

To quantify PHB, we used direct acid extraction-methylation [46, 47], wherein PHB is extracted, depolymerised and converted to its methylated monomer (3-R-hydroxybutyric acid) in one step. 200 mg of soil was digested for three hours at 100 °C with 800 µl CHCl_3_ + 800 µl 5% H_2_SO_4_ in methanol containing 0.05 mg ml^-1^ sodium benzoate as an internal standard. Digests were phase-separated with H_2_O and organic phases were dried with Na_2_SO_4_ prior to PHB quantification with GC-MS. Quantitative performance of the method was verified by spike and recovery tests showing recovery of PHB added to soil was 96.9%.

Trehalose was quantified following methods developed for similar soils [33]. Fresh soil samples ( ~5 g dry mass equivalent) were extracted in shaker-mounted ice bath (10 min at 100 r.p.m.) with 30 ml chilled 1 M KCl containing 5% CHCl_3_. These extracts notionally contain microbial along with adsorbed and free pools of trehalose; however, prior work determined that the vast majority of trehalose is intracellular for fresh, well-hydrated soils [33, 48], such that trehalose extracted via KCl-CHl_3_ in our study can be regarded as an index intracellular trehalose. Extracts were subject to two-step derivatisation of methoxymation (20 mg ml^-1^ methoxamine hydrochloride in anhydrous pyridine) followed by trimethylsilyation (N-Methyl-N-trifluoroacetamide at 37 °C for 30 minutes). Trehalose concentrations were determined via GC-MS [33].

### Soil incubation experiment

We carried out a 10-day soil incubation to examine short-term responses of soil microbial growth, respiration, and C-storage to additions of C and P. For this incubation, we used soils (0–10 cm) collected from “Basalt site 1” and “Sandstone site 2”, which respectively represent high and low fertility soils relative to our gradient (Table 1). Soils were collected in March 2022. Twenty-five grams (oven-dry basis) of fresh soil were weighed into 70 ml polypropylene jars and pre-incubated for 10 days under ambient conditions prior to application of experimental treatments. Two small holes were drilled into the lids of the jars to allow air flow. Lids were removed every 3–5 days to ensure soils were fully aerated. Two treatments were imposed: (1) addition of 10,000 µg glucose–C g soil^-1^ and (2) addition of 100 µg NaH_2_PO_4_–P g soil^-1^. With respect to the control soils, these additions correspond to ~100-fold increases in available organic C and inorganic P, respectively. Soils were maintained at 50% of water holding capacity throughout the incubation. Soil respiration was measured at days 0 [prior to treatment application], 1, 2, 3, 4, 8, and 10 using a closed system (LI-8100, LI-Cor Inc., Lincoln, Nebraska, USA). Respiration was measured prior to moisture adjustments. On day 10, MBC, PLFAs, NLFAs, and PHB were quantified as described above.

**Table 1:** Chemical and biological properties (means ± standard error) of surface soils (0–10 cm depth) at eight sites located within the West Head section of Ku-ring-gai Chase National Park, New South Wales, Australia (n = 3 in all cases, except for trehalose where n = 1 for Shale site 2, Ironstone site 2, and Sandstone site 2).

	Moisture (%)	pH (H_2_O)	Total organic C (%)	K_2_SO_4_extractable soluble organic C (µg C g soil^-1^)	Total N (µg P g soil^-1^)	Total P (µg P g soil^-1^)	Mehlich-extractable PO_4_^3-^ (µg P g soil^-1^)	Microbial C (µg C g soil^-1^)	Microbial substrate C:P ratio (mass basis)	Microbial P (µg P g soil^-1^)	Microbial C:P ratio (mass basis)	Microbial C:P ratio (molar basis)	Phosphomonoesterase activity (µg PNP g soil^-1^ h^-1^)	Phosphodiesterase activity (µg PNP g soil^-1^ h^-1^)	β-D-glucosidase activity (µg PNP g soil^-1^ h^-1^)	β-D-glucosidase activity : phosphatase activity ratio	Microbial polar lipid fatty acids (PLFAs; nmol g soil^-1^)	Bacterial PLFAs (nmol g soil^-1^)	Fungal PLFAs (nmol g soil^-1^)	Neutral lipid fatty acids (µg C g soil^-1^)	Polyhydroxybutyrate (µg C g soil^-1^)	Trehalose (µg C g soil^-1^)	Neutral lipid fatty acids (µg C g SOC^-1^)	Polyhydroxybutyrate (µg C g SOC^-1^)	Trehalose (µg C g SOC^-1^)
Basalt site 1	41.0 ± 8.5	5.62 ± 0.11	4.28 ± 0.5	102 ± 19	2577 ± 139	620 ± 91	1.96 ± 0.68	553 ± 149	59.6 ± 57.4	38.2 ± 29.8	29.4 ± 17.9	75.6 ± 46.3	952 ± 265	296 ± 41	205 ± 36	0.11 ± 0.09	67.0 ± 10.5	42.7 ± 7.4	9.5 ± 0.9	22.3 ± 1.5	33.4 ± 4.1	0.9 ± 0.1	532 ± 61	807 ± 156	22.1 ± 2
Basalt site 2	30.6 ± 1.4	4.82 ± 0.13	3.05 ± 0.3	53 ± 13	2240 ± 97	243 ± 60	0.67 ± 0.19	273 ± 27	80.0 ± 36.4	28.5 ± 4.4	9.7 ± 1.3	25.1 ± 3.4	270 ± 24	229 ± 17	61 ± 14	0.17 ± 0.19	42.5 ± 5.9	27.4 ± 4.0	5.4 ± 0.9	10.3 ± 1.2	15.1 ± 2.0	4.3 ± 1.5	339 ± 24	514 ± 115	138 ± 40
Shale site 1	17.1 ± 1.6	4.82 ± 0.16	2.39 ± 0.2	68 ± 5	1766 ± 154	168 ± 11	1.41 ± 0.41	327 ± 54	53.6 ± 20.4	22.4 ± 2.7	14.4 ± 1.0	37.1 ± 2.5	381 ± 80	162 ± 27	229 ± 44	0.41 ± 0.23	42.1 ± 7.9	23.2 ± 4.6	7.8 ± 1.2	14.3 ± 1.8	20.5 ± 2.1	41.0 ± 1.8	603 ± 91	858 ± 77	1740 ± 192
Shale site 2	35.9 ± 9.3	5.27 ± 0.22	4.51 ± 0.8	87 ± 28	2765 ± 712	164 ± 10	2.48 ± 1.62	419 ± 150	53.2 ± 68.1	38.1 ± 10.1	10.5 ± 1.5	27.1 ± 3.8	1,155 ± 602	244 ± 87	412 ± 188	0.27 ± 0.23	46.5 ± 10.0	27.2 ± 6.1	7.0 ± 1.2	17.1 ± 3.8	25.4 ± 5.5	7.2	393 ± 78	587 ± 117	248
Ironstone site 1	16.3 ± 1.6	5.19 ± 0.26	1.2 ± 0.1	66 ± 8	750 ± 95	131 ± 9	0.03 ± 0.01	203 ± 30	2981 ± 1060	0.7 ± 0.4	417 ± 295	1,075 ± 761	513 ± 120	307 ± 31	97 ± 31	0.08 ± 0.41	15.5 ± 2.0	7.0 ± 1.1	3.5 ± 0.3	14.6 ± 1.3	36.1 ± 1.8	0.8 ± 0.2	1238 ± 145	3114 ± 450	68.3 ± 26
Ironstone site 2	15.0 ± 2.9	5.01 ± 0.14	1.41 ± 0.2	48 ± 10	1059 ± 87	119 ± 15	0.17 ± 0.12	186 ± 11	528 ± 978	3.5 ± 1.0	59.9 ± 23.7	154 ± 61	774 ± 177	282 ± 34	95 ± 37	0.11 ± 0.21	21.4 ± 1.4	8.1 ± 1.0	6.2 ± 0.5	18.4 ± 2.7	30.0 ± 1.5	12.5	1431 ± 437	2248 ± 416	686
Sandstone site 1	14.1 ± 0.6	5.00 ± 0.22	0.97 ± 0.1	57 ± 4	900 ± 184	95 ± 17	0.07 ± 0.04	156 ± 51	1266 ± 1243	3.3 ± 0.9	43.5 ± 27.0	112 ± 70	630 ± 271	245 ± 14	54 ± 10	0.13 ± 0.44	15.2 ± 2.5	6.4 ± 1.1	3.5 ± 0.6	9.9 ± 1.7	18.7 ± 1.8	1.0 ± 0.4	1016 ± 139	1924 ± 52	98.2 ± 33
Sandstone site 2	17.3 ± 2.6	5.05 ± 0.08	1.62 ± 0.4	62 ± 8	851 ± 126	36 ± 14	0.17 ± 0.08	235 ± 43	636 ± 1401	3.2 ± 0.9	78.7 ± 58.2	203 ± 150	931 ± 256	407 ± 106	160 ± 25	0.07 ± 0.14	24.1 ± 2.7	9.5 ± 1.0	6.0 ± 0.5	20.5 ± 2.1	41.0 ± 10.3	1.7	1361 ± 224	2605 ± 632	165

### Stoichiometric modelling of microbial carbon allocation in response to altered elemental resource availability

The stoichiometric model of microbial growth developed by Manzoni, et al. [8]) was parameterised for Basalt site 1 and Sandstone site 2 to predict microbial growth rates, respiration rates, and C-storage dynamics in response to changes in substrate C:P. The model describes the rates of carbon (C) and phosphorus (P) uptake, assimilation, allocation to storage, and respiration by a homogeneous microbial community. Full model details are provided in the Supplementary Information.

### Statistical analyses

Statistical analyses were carried out in R (version 4.1.1) [49]. Full details of our analyses are provided in the Supplementary Information. We used the “lme4” and “pbkrtest” packages [50, 51] to compare nested linear mixed effects models to test the significance of soil type and soil properties as predictors of microbial C-storage. In these models, PLFA-C was used as a covariate to account for collinearity between microbial storage-C and microbial biomass. In other words, our estimates of intracellular C-storage are scaled to non-storage biomass, as indexed by PLFA-C, and therefore represent the extent of intracellular “allocation” of C to storage compounds. Because PHB is synthesized only by prokaryotes, we used bacterial PLFA as the covariate for PHB analyses. Models included a random intercept term for sampling site to account for non-independence of samples collected from the same site. We used an equivalent modelling approach to compare fungal : bacterial PLFA ratios (molar basis) among soil types and to test the significance of fungal : bacterial PLFA ratios as predictors of NLFA and trehalose allocation, and Gram positive bacterial PLFA : Gram negative bacterial PLFA ratios as a predictor of PHB allocation. Microbial PLFA composition was compared among soil types using non-metric multidimensional scaling analysis (based on two dimensions and Bray-Curtis dissimilarities) and permutational analysis of variance (PERMANOVA) using “vegan” [52]. Molar quantities of each microbial PLFA were converted to relative molar quantities and log-transformed (log_e_[*x* + 1]) prior to these analyses. The effects of incubation treatments on pools and fluxes of microbial C were evaluated using factorial ANOVAs, with covariate terms for microbial PLFA-C in the case of NLFA and bacterial PLFA-C in the case of PHB.

## Results

### Variation of microbial carbon storage across a natural soil fertility gradient

Soil properties varied widely across our sites (Table 1), with basalt- and shale-derived soils containing substantially more total and “available” P, and having markedly lower C:P ratios, than soils derived from ironstone and sandstone (Fig. 2, Table 1). Soil total N ranged from 750 mg kg^-1^ in ironstone-derived soil to 2,765 mg kg^-1^ in shale-derived soil.

**Fig. 3:**
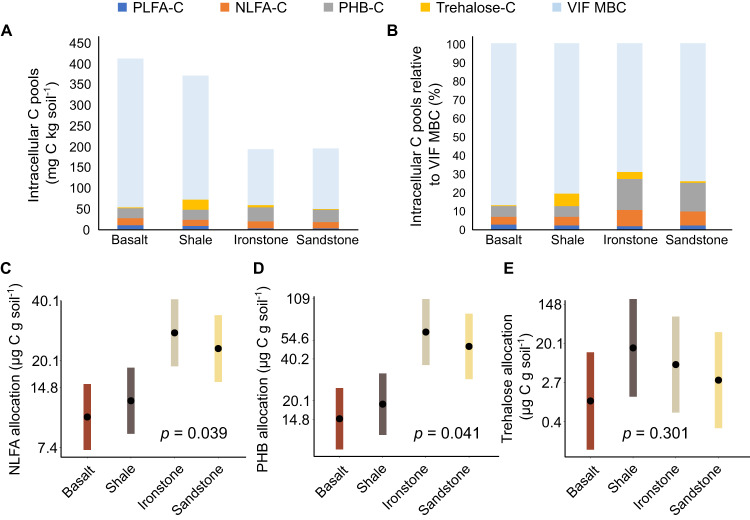
Dynamics of community-level microbial C allocation in surface soils (0–10 cm) across a natural gradient of soil fertility. **A** absolute contents of microbial polar lipid fatty acids (PLFA), neutral lipid fatty acids (NLFA), polyhydroxybutyrate (PHB), trehalose, and overall biomass C (MBC) as determined by the vacuum infiltration chloroform fumigation (VIF) method; **B** quantities of PLFA, NLFA, PHB, and trehalose presented relative to the total quantity of microbial biomass C as determined by the VIF method; and estimated marginal mean values (±95% confidence intervals) of NLFA (**C**), PHB (**D**), and trehalose (**C**) C in surface soils for a given value (the overall mean value) of polar lipid fatty acid-derived C (which serves as a proxy of non-storage microbial biomass C) according to linear mixed-effects models (*p* values indicate the significance of soil parent material as a model term; n = 24 for NLFA and PHB and 18 for trehalose; note that response values are provided on a natural log scale).

Absolute quantities of putative storage compounds differed among the sites, ranging from 9.9 ± 1.7 to 22.3 ± 1.5 µg C g soil^-1^ for NLFAs, 15.1 ± 2.0 to 41.0 ± 10.3 µg C g soil^-1^ for PHB, and 0.8 ± 0.2 to 41.0 ± 1.8 µg C g soil^-1^ for trehalose (Table 1; Fig. 3A). Together, these storage compounds corresponded, on average, to ~21% of total microbial biomass C as estimated by chloroform fumigation (Fig. 3B), although we note that the latter method may not accurately capture variable portions of hydrophobic intracellular compounds like NLFA and PHB [43, 53].

**Fig. 4:**
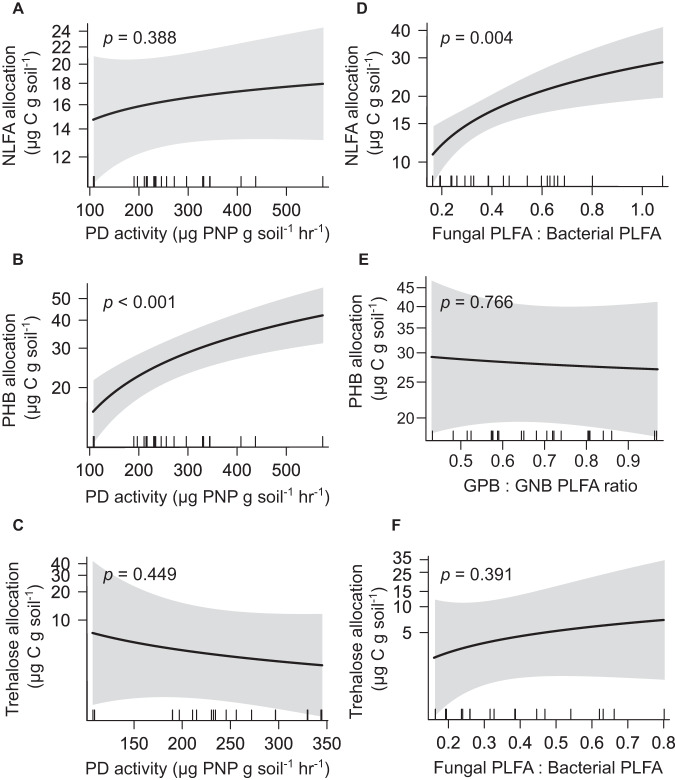
Drivers of community-level soil microbial C-storage. Community-level allocation of soil microbial carbon (C) to neutral lipid fatty acids (NLFA), polyhydroxybutyrate (PHB), and trehalose in relation to (**A**–**C**) the potential activities of phosphodiesterase in soil (“PD”; used here as an indicator of belowground biological phosphorus-demand) and (**D**–**F**) the ratios of PLFAs associated with major microbial taxa (used here as a univariate index of microbial community composition; ratios of fungal PLFA to bacterial PLFA were used in models of NLFA and trehalose allocation, whereas ratios of Gram positive bacterial PLFA to Gram negative bacterial [GNB] PLFA were used to model allocation of PHB, which is synthesised exclusively by bacteria); *n* = 24 for NLFA and PHB and 18 for trehalose; in all models, microbial PLFA-C was used as a covariate (bacterial PLFA-C in the case of PHB) to account for positive associations between absolute soil contents of non-storage microbial biomass and storage biomass. Thus, relationships presented are those predicted for a given value (the overall mean value) of microbial PLFA-C (or bacterial PLFA-C in the case of PHB). Responses are presented on a log-scale.

Quantities of NLFA standardised to total SOC differed among the fertile (95% CIs: 333–638 µg C g SOC^-1^) and infertile (912–1746 µg C g SOC^-1^) soils, and the same was true for PHB (448–893 µg C g SOC^-1^ in fertile soils versus 1633–3339 µg C g SOC^-1^ in infertile soils) but not for trehalose (18–2,145 µg C g SOC^-1^ in fertile soils versus 0–527 µg C g SOC^-1^ in infertile soils. Likewise, community-level allocation of intracellular C to NLFA and PHB (as indicated by NLFA/PHB standardised to the PLFA covariate) was greater in the infertile soils than in the comparatively fertile soils (Fig. 3C, D). Soil type was not a significant predictor of trehalose levels (Fig. 3E). However, no indices of soil C, N, or P were meaningful predictors of microbial storage allocation. Activities of β-D-glucosidase and phosphatases were not significant predictors of NLFA or trehalose allocation (Fig. 4A, C), while phosphomonoesterase and phosphodiesterase activities were significant positive predictors of PHB allocation by bacteria (e.g., Fig. 4B). Meanwhile, NLFA allocation scaled positively and significantly with fungal : bacterial PLFA ratios (Fig. 4E), whereas PHB and trehalose allocation were unrelated to PLFA ratios (Fig. 4D, F).

**Fig. 5:**
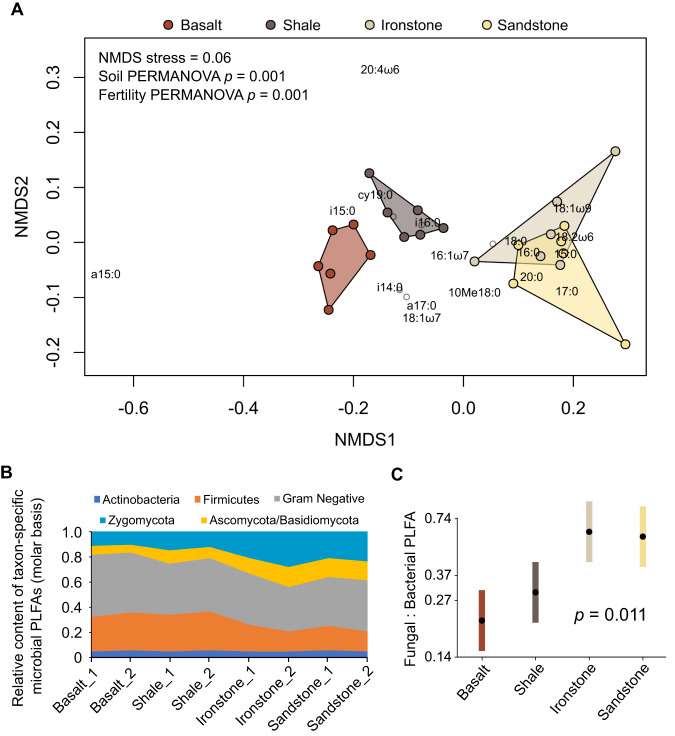
Soil microbial community composition across a natural gradient of soil fertility. **A** two-dimensional non-metric multidimensional scaling analysis of relative molar concentrations of microbial polar lipid fatty acids (PLFAs; n = 24; permutational analysis of variance [PERMANOVA] *p* values indicate the significance of soil type and soil fertility category as sources of variation in PLFA composition); **B** relative content of taxon-specific microbial PLFAs; **C** estimated marginal mean values (±95% confidence intervals) of log-transformed ratios of fungal PLFA content to bacterial PLFA content (calculated on a molar basis), with *p* values indicating the significance of soil type as a model term (n = 24; note that responses are on a log scale). PLFA nomenclature and taxonomic assignations following Joergensen (2022).

Microbial PLFA composition differed among the soil types (*p* = 0.001; Fig. 5A). Fertile soils had higher relative abundances of Firmicutes and Gram-negative Bacterial biomarkers, whereas fungal biomarkers had higher relative abundances in the infertile soils. Most variation in PLFA composition occurred at the Kingdom level (Fig. 5B, Fig. S1), with fungal PLFA : bacterial PLFA ratios 2–3-fold higher in sandstone- and ironstone-derived soils than in basalt- and shale-derived soils (Fig. 5C).

### Short-term responses of microbial growth, carbon-allocation, and community composition to experimental alterations of carbon and phosphorus availability

**Fig. 6:**
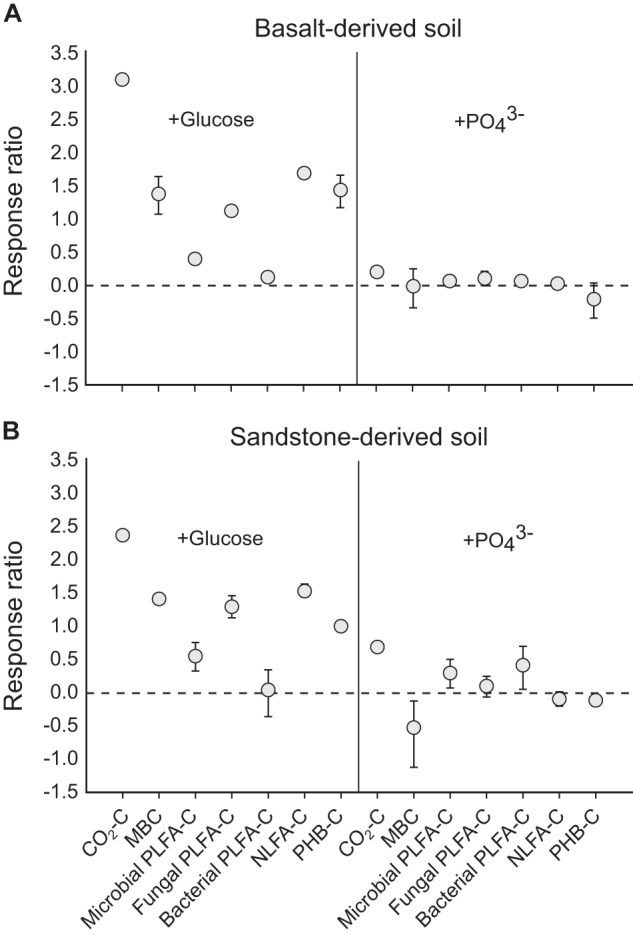
Responses of soil carbon (C) pools and fluxes to altered substrate stoichiometry. Panels summarize the effects of glucose and PO_4_^3-^ (as NaH_2_PO_4_) addition on soil CO_2_ respiration (integrated over 10 days of incubation), microbial biomass C (MBC), total microbial polar lipid fatty acid-derived C (PLFA-C), fungal PLFA-C, bacterial PLFA-C, absolute content of neutral lipid fatty acid-derived C (NLFA-C), and absolute content of polyhydroxybutyrate-derived C (PHB-C) for (**A**) a basalt-derived soil and (**B**) a sandstone-derived soil. Soil C pools were measured after 10 days of incubation. Effects are expressed as mean response ratios (log_e_[*X̄*_*treatment*_ / *X̄*_*control*_]) ± 95% confidence intervals (n = 5). Note that there were no scaling covariates used in the calculation of response ratios. Instead, absolute contents of each C pool (ug C g soil^-1^) were used. We consider effects statistically significant where confidence intervals do not overlap with zero (i.e., the horizontal dotted line). Detailed treatment comparisons for individual response variables based on linear models with scaling covariates for non-storage biomass are provided in Supplementary Fig. S2.

Addition of glucose and PO_4_^3-^ had varied effects on microbial C allocation after ten days of incubation (Figs. 6 and S2). Soil MBC was ~4-fold higher in glucose-amended soils than in controls (Figs. 6 and S2a). Similarly, total microbial PLFA increased with addition of both glucose and PO_4_^3-^ (*p* < 0.0001), with the effect of glucose larger than that of PO_4_^3-^ (Figs. 6 and S2b). However, the effect of glucose on total microbial PLFAs was due largely to fungi, rather than bacteria, as total bacterial PLFA was not affected by glucose (Figs. 6 and S2c, d). By contrast, total bacterial PLFA was increased by PO_4_^3^, but only for the sandstone-derived soil (soil × treatment *p* = 0.015; Figs. 6s and S2d), while PO_4_^3-^ had no effect on total fungal PLFA (Figs. 6 and S2c). The composition of microbial PLFAs differed markedly among the incubation treatments due to the strong effect of glucose on fungal PLFAs (Fig. S3). Glucose increased the absolute contents and allocation (i.e., contents standardised to the mean value of PLFA-C) of intracellular C to NLFA and PHB for both soils (Figs. 6 and S2e–h). Addition of PO_4_^3-^ had no effect on NLFA or PHB (Figs. 6 and S2e, f).

Respiration rates were greater for the basalt-derived soils (454 ± 9.6 µg CO_2_–C g soil^-1^ respired) than for the sandstone-derived soils (201 ± 8.5 µg CO_2_–C g soil^-1^ respired; Fig. S4). For both soil types, respiration rates increased dramatically with glucose addition (Figs. 6 and S4). The addition of PO_4_^3-^ did not significantly affect CO_2_ efflux from the basalt-derived soil, but induced an approximate doubling of CO_2_ efflux from the sandstone-derived soil (Figs. 6 and S4).

### Microbial growth and carbon allocation as predicted by a stoichiometric model

**Fig. 7:**
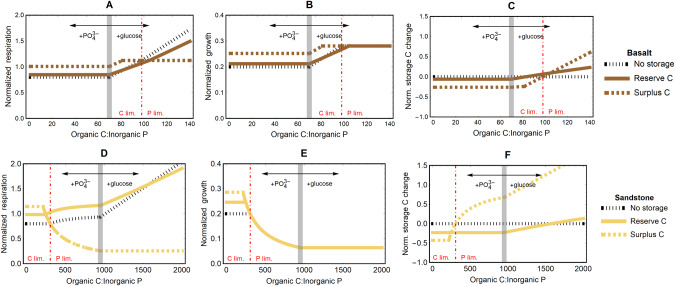
Predictions of a stoichiometric model of microbial growth and C allocation [8] applied to two soil types from the soil fertility gradient at West Head in eastern Australia. Panels show effects of changes in organic C to inorganic P ratio on: **A**, **D**) microbial growth; **B**, **E** microbial respiration; and **C**, **F** net intracellular C-storage changes in two soils developed on different parent material: **A**–**C** a basalt-derived soil and **D**–**F** a sandstone-derived soil. In each panel, curves are color-coded according to the mode of C-storage use (reserve vs. surplus); the black dotted curves refer to microbes that do not have intracellular storage; the gray thick vertical lines indicate the organic C:inorganic P ratio in the sampled soils; the red dot-dashed vertical lines indicate the threshold element ratios (i.e., organic C:inorganic P ratio at which microbes switch from C (to the left) to P limitation (to the right)), calculated as ratios of microbial C:P over growth efficiency. Organic C:inorganic P ratios are changed by increasing organic C at fixed P for C:P higher than the baseline value, or increasing inorganic P at fixed C for C:P lower than the baseline value. All rates on the y-axes are normalized by the C uptake rate at the baseline conditions. To parameterise the model runs, we chose as a baseline the extractable C:PO_4_^3-^ ratios of the basalt- and sandstone-derived soils (Table 1; gray vertical lines).

When the stoichiometric model was parameterised for the basalt-derived soil, addition of PO_4_^3-^ had no effect on respiration, growth, or net C-storage, regardless of microbial C-storage capacity (Fig. 7A–C). Addition of C increased respiration, growth, and net C-storage until microbial biomass switched from C-limitation to P-limitation. Adding further C under P-limitation increased microbial respiration, but only where C-storage was absent or serving the reserve mode (Fig. 7A), whereas growth did not increase (Fig. 7B) because excess C was respired. Excess C was instead allocated to storage in the surplus storage mode, resulting in stronger increases in surplus storage compared to reserve storage (Fig. 7C).

When the model was parameterised for the sandstone-derived soil, the addition of PO_4_^3-^ had complex effects on all variables, with these effects dependent on the ability of microorganisms to allocate C to either surplus or reserve storage (Fig. 7D–F). Respiration and C-storage were unaffected by PO_4_^3-^ addition, while growth increased, in scenarios in which C-storage was absent or served the reserve mode. When storage served the surplus mode, respiration and growth increased with PO_4_^3-^ addition, while C-storage declined, until the microbial biomass transitioned from P-limitation to C-limitation. Addition of C to the sandstone-derived soil increased respiration where C-storage was absent or serving the reserve mode, but respiration was unaffected by C addition where C-storage served the surplus mode (Fig. 7D, E). Growth was not affected by C addition, because P-limited microbes only grow when P is added (Fig. 7E). Allocation of C to storage increased with C addition regardless of storage mode, but the increase was faster for the surplus mode (Fig. 7F).

## Discussion

Ours is the first study to characterise microbial C-storage dynamics across both natural and experimental soil fertility gradients and, by doing so, investigate hypothetical links between storage dynamics, resource availability, microbial resource demand, and microbial community composition. Absolute contents of storage compounds in our samples (Table 1) were broadly similar to prior studies, which indicate a typical range of 2–20 µg C g soil^-1^ for NLFA [29, 31, 32], 1–18 µg C g soil^-1^ for PHB [28, 30, 32], and 2–32 µg C g soil^-1^ for trehalose [[33], Hussain and Warren, unpublished data]. However, when normalised to total SOC, values from representative prior studies equate to ~58–139 µg NLFA-C g SOC^-1^ [29] and ~14.6–307 µg PHB-C g SOC^-1^ [30]. Our values are up to an order of magnitude higher, averaging 864 ± 205 [95% CI] µg NLFA-C g SOC^-1^ and 1582 ± 431 µg PHB-C g SOC^-1^. One potential explanation for this difference is the infertile nature of our study landscape. By global standards, even the “fertile” soils in our gradient have low levels of P and other nutrients (Table 1). Such conditions are likely conducive to extensive synthesis of storage compounds across the entire study area. Given that our gradient consisted of just four true “points” at the lower end of the fertility spectrum, future studies should consider expanding the number of soil types and consider other gradients, such as climatic gradients and soil developmental chronosequences.

Although our gradient effectively ranged from low to extremely low fertility, this range was sufficient to drive substantial variation in allocation of microbial C to NLFA and PHB (with allocation indicated by the amount of storage standardised to the mean value of PLFA-C); Fig. 3A–D). Analogous effects were observed for NLFA and PHB reported on the basis of total SOC (Table 1). We conclude, therefore, that soil fertility is an important driver of microbial C-storage via NLFA (i.e., TAGs) and PHB across our study landscape, and that microbial communities associated with infertile soils allocate more C to NLFA and PHB than those associated with comparatively fertile soils. Notably, NLFA and PHB together comprised ~85% of the stored C we measured (Table 1). Ancillary data for Sandstone site 1 and Basalt site 1 suggest an absence of wax esters and cyanophycin in soil (results not shown). Thus, NLFA and PHB are likely the dominant C-storage compounds synthesized by soil microorganisms at West Head, such that our conclusion can reasonably be generalised to intracellular microbial C-storage *en masse*. It is possible, however, that glycogen—a hydrophilic polymer of glucose that occurs across bacteria and fungi [7, 54]—makes an as-yet unquantified contribution to intracellular C-storage. Likewise, we assume that storage compounds originate entirely from living cells. This is supported by the rapid turnover of lipids and trehalose observed in studies of similar soils [33, 55, 56] and the rapid synthesis of NLFA and PHB seen with ^13^C-labelled glucose addition elsewhere [30, 32]. Nevertheless, we cannot discount that some fraction of storage-C came from microbial necromass. Glycogen dynamics and differentiation of storage in biomass versus necromass should be priorities for future research in this area.

Our finding that PHB allocation was strongly positively correlated with potential phosphatase activity (Fig. 4B) suggests that community-level allocation of C to PHB by bacteria was coupled with belowground biological P-demand—and potentially with bacterial P-deficiency – across our fertility gradient. Indeed, culturing studies have shown that nutrient deprivation triggers PHB synthesis, with PHB readily serving as a intracellular sink for excess C [17–19, 57]. Based on this result, along with our finding that bacterial PLFAs increased with PO_4_^3-^ addition to the sandstone-derived soil (Figs. 6 and S2d), we suggest that increasing PHB allocation from high to low fertility soil represents enhanced surplus C-storage by increasingly P-limited bacteria. This is further supported by the disconnect between Gram positive : Gram negative bacterial PLFA ratios and PHB allocation (Fig. 4E), given that surplus storage can vary without accompanying shifts in microbial community composition, and by the positive response of PHB to glucose with no concomitant increase in bacterial PLFA (Fig. 6). Only the variable portion of PHB need serve the surplus mode in this scenario, with a large proportion of PHB potentially allocated constitutively as reserve C-storage. However, PHB dynamics were not related to soil P indices, and plants and fungi likely contributed to the phosphatase activities we measured. Moreover, in theory, provision of P to P-limited microorganisms should trigger a decline in intracellular surplus C-storage as organisms rapidly reallocate stored C to structural tissues during growth [8], yet we did not observe this for PHB (Fig. S2f). Speculative reasons for this include a large proportion of reserve C-storage by bacteria that overshadows short-term dynamics of surplus mode PHB, and/or additional roles for PHB, beyond that of C-storage, in natural environments. Additional work is required to fully ascertain the specific role of bacterial P nutrition a driver of PHB dynamics in soil bacteriomes.

Community-level NLFA allocation was related to microbial community composition (i.e., fungi : bacteria ratios; Fig. 4D), with the corollary that NLFAs (or TAGs) mostly served as reserve C-storage for C-limited fungi [7, 35]. This is consistent with our initial predictions (Fig. 1), and aligns with the recent finding that TAGs are an important form of reserve C-storage in soil microbial communities [32]. Prior studies at other locations in Australia have shown a similar trend toward high fungal : bacterial PLFA ratios in low fertility soils [58, 59], so the relationship between soil fertility and community-level microbial NLFA allocation is likely widespread. Additional support for the view that taxonomic shifts drive community-level NLFA allocation was provided by our incubation experiment, in which increases in NLFA following glucose addition were paralleled by large increases in total PLFA for fungi but not bacteria (Fig. 6). Thus, the positive effects of glucose on NLFA allocation were likely driven by increases in the abundance/biomass of fungal taxa with inherently high constitutive allocation of C to NLFA.

According to our stoichiometric modelling (Fig. 7), the effects of glucose additions were more compatible with reserve C-storage than surplus C-storage [8]. Indeed, according to the model, a substantial increase in respiration with glucose addition is largely incompatible with surplus storage, because C in excess of requirements should be stored rather than respired [7, 8]. However, in our experiment, respiration increased markedly with glucose for both soils (Figs. 7 and S4). If microbial storage served purely the surplus mode, the model predicts no change in “structural” (i.e., non-storage) biomass following glucose addition (Fig. 7B, E), but in fact there was, as evidenced by our PLFA results (Fig. 6). For our data to match theory, it is therefore necessary that the increases in C pools and fluxes we observed in response to glucose were driven by (initially) C-limited microorganisms. Indeed, running the model parameterized for the sandstone-derived soil with a higher microbial C:P representing C-limited fungi results in a positive response of growth to glucose (results not shown), similar to the results for basalt-derived soil. This supports our view that a proliferation of C-limited fungi with substantial constitutive allocation to reserve-mode NLFA could underpin the glucose responses in our incubation. In our interpretation of model results, we have focused on our incubation experiment (Figs. 6 and S2), as it explicitly isolates substrate C:P from other variables. However, the model is agnostic as to whether storage dynamics are driven by plasticity of individuals or differences in community composition [8]. Meanwhile, our empirical results indicate diverging C-allocation strategies within soil microbial communities, with a large portion of C-storage dynamics likely underpinned by compositional shifts across natural and experimental substrate stoichiometry gradients. Thus, there is potential to enhance storage-explicit models of microbial C and nutrient cycling by allowing for diverging C-storage strategies among major microbial taxa. This would also allow modelling different nutrient re-cycling strategies, such as efficient retention of nutrients at senescence [60], that are not accounted for in the current model.

The ability of microorganisms to store C is predicted to enhance microbial nutrient and C use efficiency [7, 8], with implications ranging from local- to global-scales. Locally, the enhanced microbial P retention associated with C-storage (and particularly surplus storage as PHB by bacteria [8]) should mean more P sequestered within microbial biomass and less PO_4_^3-^ entering the soil solution via mineralisation. Given that levels of plant-available P in sandstone-derived soils at West Head are among the lowest reported globally [61], the conservative microbial P cycling enabled by intracellular C-storage could contribute to sustained ecosystem productivity [26] and persistence of plant species adapted to P-deficient soils [62, 63]. Globally, the enhanced microbial CUE theoretically associated with intracellular C-storage ability [8] could contribute to accumulation of soil organic C [4–6]. However, at our sites, SOC stocks in surface soil were ~9.8–16 tonnes C ha^-1^ in infertile soils versus ~27–44 tonnes C ha^-1^ in fertile soils. Thus, the greater allocation of C to intracellular storage by microorganisms in infertile soils was not paralleled by greater bulk SOC storage. Storage compounds were detected in all soils (Table 1), so it may be the ability to storage C, rather than the extent of C-storage, that influences microbial CUE. It is also possible that a substantial fraction of intracellular C-storage served the reserve mode, which is expected to have smaller impacts on CUE than the surplus mode [8]. Additional work is needed to explore links among intracellular C-storage, CUE, and bulk SOC storage.

Our findings indicate that community-level microbial C-storage varies across natural and experimental soil fertility gradients, with this variation likely controlled by a hierarchy of physiological and community-level mechanisms (Fig. 1). PHB allocation was positively coupled with belowground P-demand under steady state conditions and was seemingly unrelated to broad bacterial community composition based on PLFA biomarkers, with at least some PHB serving the theoretical surplus C-storage mode for P-limited bacteria. In contrast, NLFA allocation increased with fungal : bacterial ratios across both natural and experimental soil fertility/substrate C:P gradients, and likely represented reserve C-storage for C-limited fungi. Nevertheless, PHB and NLFA showed similar dynamics, leading to a substantially larger pool of intracellular C-storage in low fertility and/or high substrate C:P soils for a given amount of non-storage microbial biomass. Thus, our study provides the first account of a predictable synergism between community-level and individual-level drivers of C-storage dynamics in soil microbial communities.

### Supplementary information

Supplementary Material

## Data Availability

The datasets generated during and/or analysed during the current study are available in the Zenodo repository (10.5281/zenodo.8412105).
