# Supplementary material for: Community composition and physiological plasticity control microbial carbon storage across natural and experimental soil fertility gradients
Source: ISME J. 2023 Oct 18;17(12):2259–69. doi: 10.1038/s41396-023-01527-5 (PMC10689824; doi:10.1038/s41396-023-01527-5)
Supplement: Supplementary file 1 — Supplementary Material [file 41396_2023_1527_MOESM1_ESM.pdf]

Supplementary Information

Supplementary Materials and Methods

Measurement of basic soil chemical and biological properties

Fresh soil samples were used for all soil chemical and biological properties, except for soil total P which was measured on air-dried soil samples. Soil was sieved at 2 mm prior to measurement of basic chemical and biological properties. Soil pH was measured with a pH probe (1:5 soil : water ratio). Biologically available soil PO_4^{3}–P was measured via molybdenum-blue spectrophotometry following extraction with the Mehlich–3 extractant and filtration with Whatman 42 filter paper [1, 2]. Total soil P was measured via molybdenum-blue spectrophotometry after digesting air-dried and finely ground soil samples in concentrated H_2SO_4 and H_2O_2 [1, 2]. Total N (as NH_4^+) was measured in the same H_2SO_4 digests through colorimetric methods [3]. Total soil organic carbon was measured via the Walkley-Black method [2].

Microbial biomass C and P were measured using the vacuum-infiltration chloroform fumigation method [4, 5]. Soils were fumigated within 48 hours of initial collection. Total soluble organic C concentrations of 0.5M K_2SO_4 extracts of fumigated and non-fumigated soil samples were quantified via spectrophotometry after digestion with potassium dichromate [6]. Total PO_4^{3-}–P concentrations of 0.5M $NaHCO_3$ of fumigated and non-fumigated soil samples (and PO_4^{3-}–P spike and recovery samples) were quantified with molybdenum-blue spectrophotometry. Concentrations of MBC and MBP were then calculated based on the difference between fumigated and non-fumigated samples using the conversion factors of 2.64 and 0.4 respectively [4, 5]. Soluble organic C in non-fumigated soil extracts serve as our index of labile organic C that is readily available to soil micro-organisms. The potential activities of three extracellular enzymes (β-D-glucosidase, ‘acid’

phosphomonoesterase, and phosphodiesterase) were measured to serve as indices of overall soil microbial C- and P-demand [7]. β-D-glucosidase hydrolyses terminal, non-reducing β-D-glylcosyl residues, resulting in the release of β-D-glucose for subsequent uptake by micro-organisms. Phosphomonoesterase and phosphodiesterase respectively hydrolyse single and double ester-bonded P to release PO_4^{3-}. The potential activities of β-D-glucosidase, acid phosphatase, and phosphodiesterase were assayed using *p*-nitrophenol spectrophotometry [8–10].

Assignation of PLFAs to microbial taxa:

Our assignation of PLFAs to microbial taxa followed Joergensen 2022 [11]. Where present, PLFAs i14:0, i15:0a, i16:0a, i17:0, i18, a15:0, a16:0, a17:0, a18:0, a19:0, 10Me16:0, 10Me17:0, 10Me18:0, cy17:0, cy19:0, 16:1ω7, 16:1ω9, 17:1ω8, and 18:1ω7 were assigned to bacteria specifically, while PLFAs 16:1ω5c, 18:1ω9c, 18:2ω6c, and 18:3ω6,9,12 were assigned to fungi. The combination of bacterial and fungal PLFAs plus unspecific microbial PLFAs (14:0, 15:0, 16:0 d, 17:0, 18:0, 20:0, 20:4ω6,9,12,15) was used to represent total microbial PLFA in our samples.

Statistical analyses

We first tested the significance of soil type and variables related to soil fertility and microbial C and P demand as predictors of NLFA, PHB, and trehalose, while using PLFA-C as a covariate to account for collinearity between microbial storage-C and microbial biomass. We used the '*lme4*' package [12] to construct nested linear mixed effects models of each storage compound. Models containing the predictor of interest were compared to equivalent, nested null models that lacked the predictor of interest using *F*-tests in which degrees of freedom were approximated by the Kenward-Roger method [13]. Base null models consisted solely of a random intercept term for sampling site in order to account for non-independence of

samples collected from the same site. The significance of total microbial PLFA-C as a covariate (total bacterial PLFA-C in the case of PHB, which is not synthesised by eukaryotes) was tested against these null models. PLFA-C was a significant, positive predictor of each putative storage compound ($P < 0.05$ in all cases). Thus, predictors of interest (soil type and continuous variables related to soil C and P availability and microbial C and P demand) were subsequently tested against models containing terms for sampling site as a random intercept and PLFA-C as a covariate. This approach enables conservative estimation of the effects of soil C and P availability and soil microbial C and P demand on the tendency for soil micro-organisms to allocate C to storage compounds while accounting for the overall influence of site and the absolute quantity of microbial biomass C. The use of PLFA-C as a covariate is conceptually similar, but statistically preferable [14–16], to the use of neutral lipid : polar lipid ratios employed in studies of fungal allocation of C to storage structures [17, 18].

Next, to understand better the potential influence of microbial community composition as a factor influencing the dynamics microbial C-storage across our soil fertility gradient, we used an equivalent modelling approach to test the significance of fungal PLFA : bacterial PLFA ratios (molar basis) as predictors of NLFA and trehalose allocation, and Gram positive (Firmicutes + Actinomycetes) bacterial PLFA : Gram negative bacterial PLFA ratios as a predictor of PHB allocation. We subsequently compared fungal PLFA : bacterial PLFA ratios among soil types using linear mixed effect models, along with overall microbial PLFA composition using non-metric multidimensional scaling analysis (based on two dimensions and Bray-Curtis dissimilarities) and permutational analysis of variance (PERMANOVA) using 'vegan' [19]. Molar quantities of each microbial PLFA were standardised (i.e., converted to relative molar quantities) and log-transformed ($\log_e[x+1]$) prior to these multivariate analyses, which were carried out on the level of individual soil samples (n = 6 for each soil type; 24 observations in total).

The effects of incubation treatments on total MBC and the molar contents of microbial, bacterial, and fungal PLFA biomarkers after ten days of incubation were evaluated using factorial ANOVAs. The effect of incubation treatments on NLFA and PHB were likewise assessed using factorial ANOVAs with an additional covariate term for total microbial PLFA-C in the case of NLFA and total bacterial PLFA-C in the case of PHB. The response of soil respiration was analysed with a factorial ANOVA that included a term for incubation day as a numeric variable. There was a significant three-way interaction between soil type, amendment, and incubation day, due to marked differences in the temporal dynamics of CO_2 respiration under glucose-addition between the basalt-derived and sandstone-derived soil. Thus, to simplify interpretation, we re-analysed respiration for each soil type separately, and we focus on those results hereafter.

Stoichiometric model of microbial carbon allocation

Full details of this model are provided in Manzoni, et al. (2021 [ref. 19]). In the model, C is partitioned between growth and storage based on the two different storage use modes. In the reserve storage mode, C that is taken up by micro-organisms is converted to new biomass or storage according to a fixed proportion, and C in excess of stoichiometric requirements is respired (overflow respiration); stored C is remobilized according to prescribed kinetics. In the surplus mode, C that is taken up is preferentially allocated to storage when it is in stoichiometric excess with respect to P; stored C is then remobilized when the external supply of organic C is lower than the stoichiometric requirements. In both modes, P is only immobilized from the inorganic P compartment and P in excess is mineralized. Microbial respiration includes both growth and maintenance components (the latter is neglected here for simplicity), along with overflow respiration to compensate stoichiometric imbalances.

The main assumption of this model is that microbial biomass must grow at a fixed 'structural' C:P ratio, but it can vary its overall biomass composition by means of intracellular C storage. In the model, by balancing C and P allocation so that the 'structural' biomass C:P is fixed, all the C and P transfer rates can be calculated as a function of the substrate C and P contents. For the purpose of this work, we consider an organic C substrate and inorganic P, whose relative proportions are changed to explore how adding organic C or inorganic P alters microbial C allocation. Moreover, we focus on storage of C only (i.e., potential P-storage is not considered). The model is only used to describe the rates of C and P transfer between the substrate and microbial compartments for given compartment sizes. In this way we simulate how a perturbation in the substrate elemental composition affects the fluxes in the short-term time span of a laboratory experiment.

Table S1. Model parameters (see detailed equations and explanations in Manzoni et al,. 2021 [ref. 19]) and measured compartment contents and C:P ratios (averages of two sites for each parent material, from Table 1).

Parameter	Parameters specific for sampled soils		Units	Source or explanation
	Basalt-derived soil	Sandstone-derived soil		
Extractable C, C_S	77.5	59.5	µg C g^{-1}	Table 1
PO_4^{3-}, P_I	1.315	0.12	µg P g^{-1}	Table 1
Extractable C:PO_4^{3-}, $C_S\!:\!P_I$	69.8	951	g C g P^{-1}	Table 1
Microbial biomass C, C_B	413	195.5	µg C g^{-1}	Table 1
Microbial storage C, C_{ST}	43.2	46.4	µg C g^{-1}	Table 1
Microbial C:P, $(C\!:\!P)_B$	19.6	61.1	g C g P^{-1}	Table 1
C:P imbalance, $\frac{C_S:P_I}{(C:P)_B}$	3.6	15.6	-	Calculated from $C_S\!:\!P_I$ and $(C\!:\!P)_B$
	Common parameters			
Microbial growth efficiency, *e*	0.2		-	Chosen value
Organic C uptake rate constant, k_S	1		d^{-1}	Chosen value
PO_4^{3-} immobilization rate constant, k_I	1		d^{-1}	Assumed = k_S
Storage C remobilization rate constant, $k_{C_{ST}}$	1		d^{-1}	Assumed = k_S
Half-saturation constant for substrate inhibition of C_{ST} remobilization, $K_{C_{ST}}$	70		µg C g^{-1}	Assumed ≈ average C_S across sites
Fraction of C allocated to storage, σ_C	0.3		-	Chosen value (only for reserve storage mode)

Supplementary Results

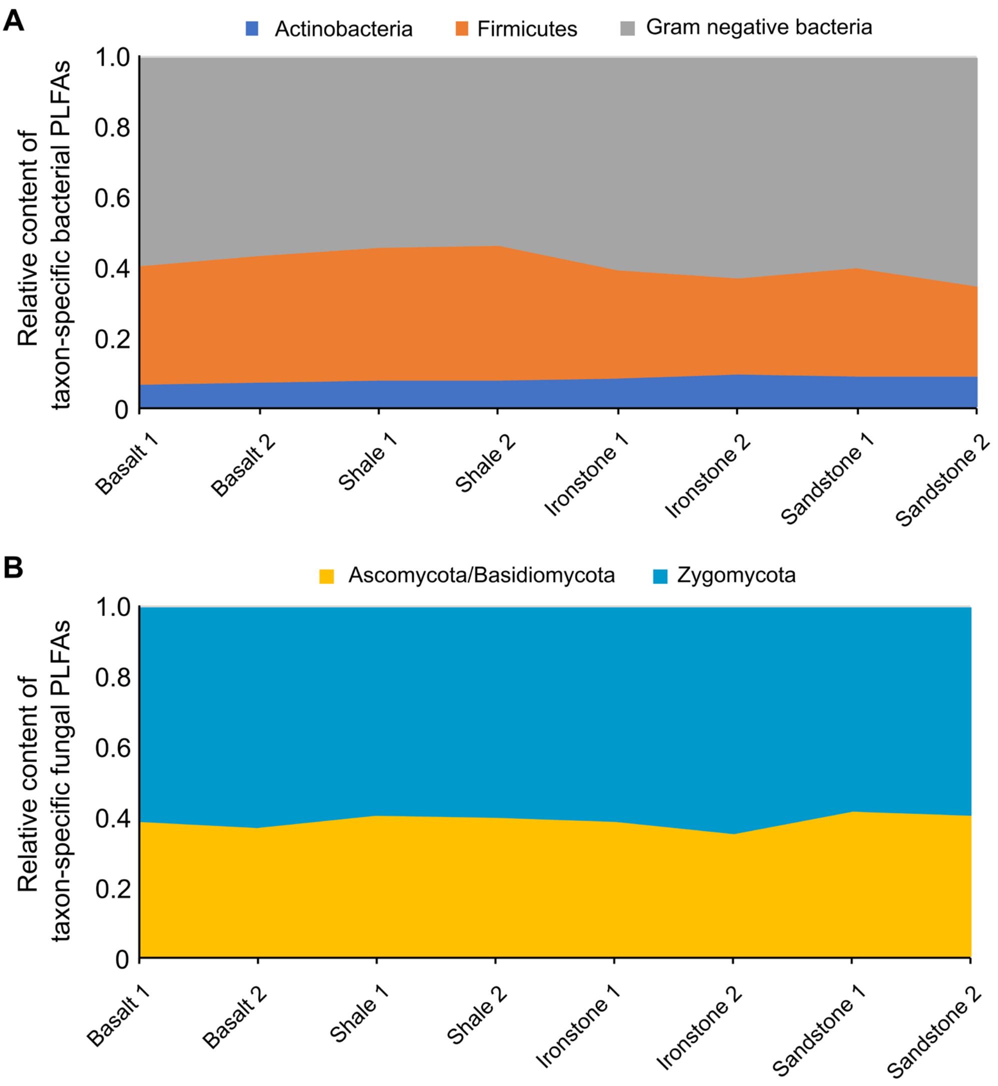

Figure S1. Relative contents of taxon-specific (a) bacterial polar lipid fatty acids (PLFAs) and (b) fungal PLFAs (molar basis) extracted from surface (0–10 cm) soils with differing parent materials at West Head, NSW, Australia, in February 2022. Taxonomic assignations of PLFAs follows Joergensen (2022 [ref. 10]).

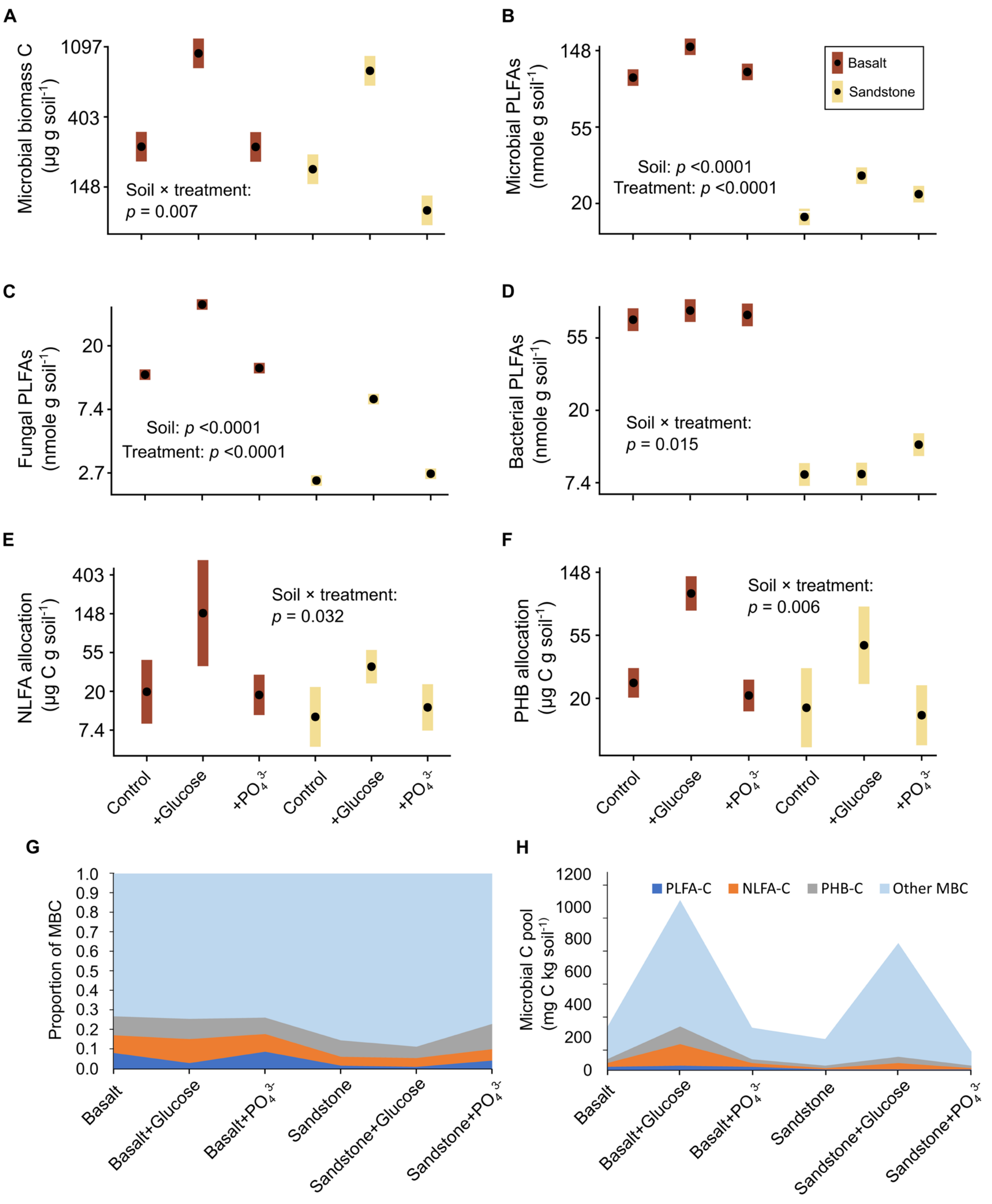

Figure S2. Estimated marginal mean values (±95% confidence intervals) of (a) microbial biomass carbon (C), (b) total microbial polar lipid fatty acids (PLFAs), (c) bacterial PLFAs, (d) fungal PLFAs, (e) neutral lipid fatty acid (NLFA)-derived C for a given value of PLFA-C (which serves as a proxy of non-storage microbial biomass C), and (f) polyhydroxybutyrate (PHB)-derived C for a given value of PLFA-C after ten days of incubation with added glucose or PO_4^{3-} (added as NaH_2PO_4) for basalt- and sandstone-derived soils (*P*-values show significant sources of variation in log-transformed response variables according to linear models; n = 5 for each treatment combination; 30 observations in total), and (g) proportional and (h) absolute levels of storage compound-derived C in basalt and sandstone soils under the respective incubation treatments. Note that y-axes on panels 'a'– 'f' are on a log scale. Legend in panel 'b' applies to panels 'a'– 'f', legend in panel 'h' applies to panels 'g' and 'h'.

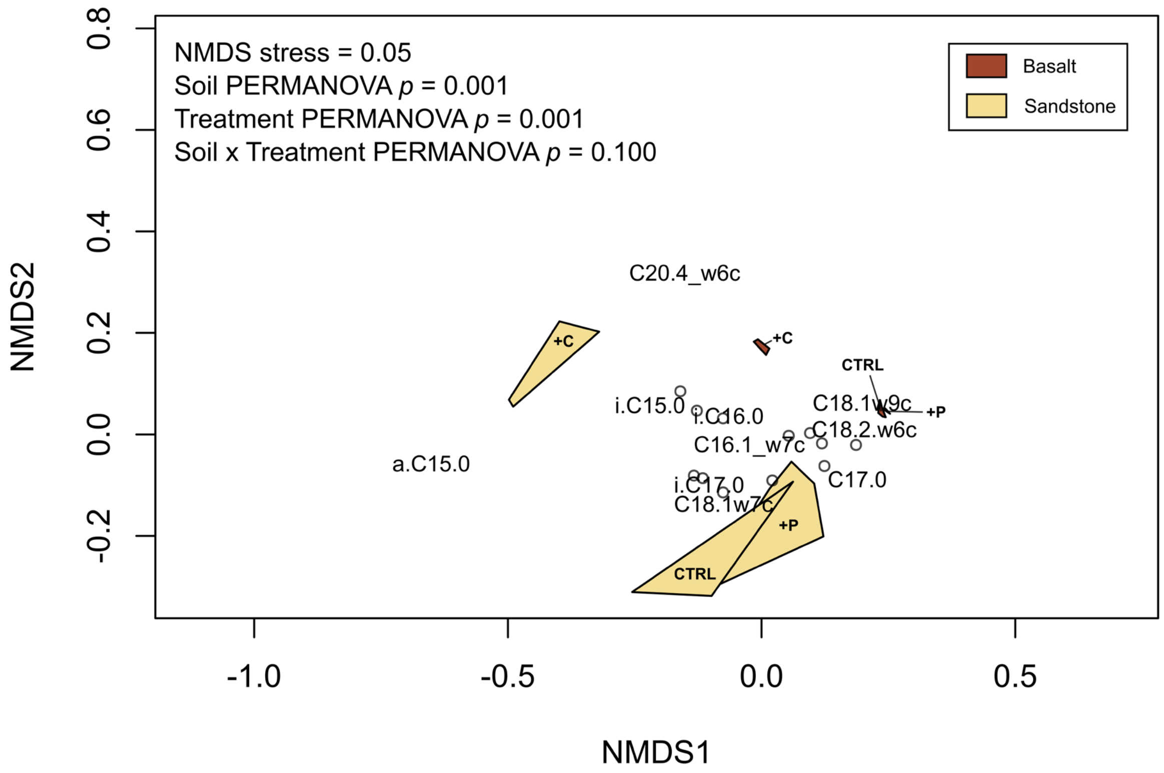

Figure S3. Two-dimensional non-metric multidimensional scaling analysis of polar lipid fatty acid (PLFA) composition (based on relative molar concentrations) of individual samples of soils used in an incubation experiment in which a sandstone-derived soil and a basalt-derived soil were subjected to additions of carbon (as glucose; +C) and phosphorus (as NaH_2PO_4; +P). Soil samples were collected from West Head, NSW, Australia in March 2022. Permutational analysis of variance (PERMANOVA) P values indicate the significance of soil type and incubation treatment as sources of variation in PLFA composition (n = 5 for each soil type × treatment combination). PLFA composition provides insight into the taxonomic composition of the living soil microbial biomass, with PLFA nomenclature and taxonomic assignations following Joergensen (2022).

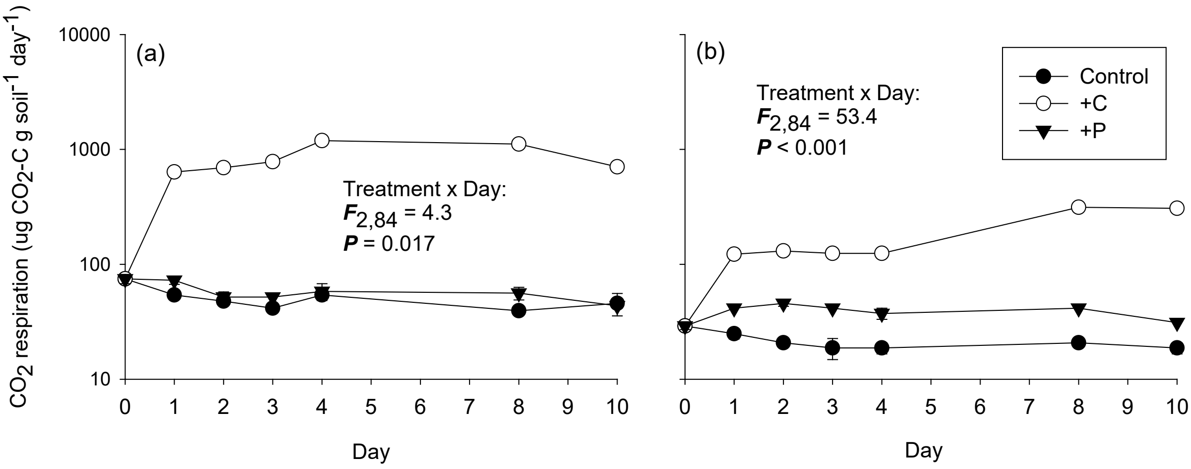

Figure S4. Rates of heterotrophic CO_2 efflux from (a) a basalt-derived soil and (b) a sandstone-derived soil in response to additions of carbon (C; as glucose) and phosphorus (P; as NaH_2PO_4) over ten days of incubation (*F*-statistics and *P*-values are from ANOVAs of CO_2 efflux rates, with n = 5 for each treatment × day combination). The two soils were analysed separately.

Supplementary references

1. Murphy J, Riley J. A modified single solution method for the determination of phosphate in natural waters. *Anal Chem ACTA* 1962; **27**: 31–36.
2. Rayment GE, Lyons DJ. Soil Chemical Methods - Australasia. *Soil Chemical Methods - Australasia* . 2019.
3. Baethgen WE, Alley MM. A manual colorimetric procedure for measuring ammonium nitrogen in soil and plant kjeldahl digests. *Commun Soil Sci Plant Anal* 1989; **20**: 961–969.
4. Vance ED, Brookes PC, Jenkinson DS. An extraction method for measuring soil microbial biomass C. *Soil Biol Biochem* 1987; **19**: 703–707.
5. Brookes PC, Powlson DS, Jenkinson DS. Measurement of microbial biomass phosphorus in soil. *Soil Biol Biochem* 1982; **14**: 319–329.
6. Cai Y, Peng C, Qiu S, Li Y, Gao Y. Dichromate digestion-spectrophotometric procedure for determination of soil microbial biomass carbon in association with fumigation-extraction. *Commun Soil Sci Plant Anal* 2011; **42**: 2824–2834.
7. Sinsabaugh RL, Hill BH, Follstad Shah JJ. Ecoenzymatic stoichiometry of microbial organic nutrient acquisition in soil and sediment. *Nature* 2009; **462**: 795–798.
8. Eivazi F, Tabatabai MA. Glucosidases and galactosidases in soils. *Soil Biol Biochem* 1988; **20**: 601–606.
9. Eivazi F, Tabatabai MA. Phosphatases in soils. *Soil Biol Biochem* 1977; **9**: 167–172.
10. Browman MG, Tabatabai MA. Phosphodiesterase Activity of Soils. *Soil Sci Soc Am J* 1978; **42**: 284–290.
11. Joergensen RG. Phospholipid fatty acids in soil—drawbacks and future prospects. *Biol Fertil Soils* 2022; **58**: 1–6.
12. Bates D, Mächler M, Bolker B, Walker S. Fitting linear mixed-effects models using lme4. *J Stat Softw* 2014; **67**: 1–48.
13. Halekoh U, Højsgaard S. A Kenward-Roger approximation and parametric bootstrap methods for tests in linear mixed models - the R package pbkrtest. *J Stat Softw* 2014; **59**.
14. García-Berthou E. On the misuse of residuals in ecology: Testing regression residuals vs. the analysis of covariance. *J Anim Ecol* 2001; **70**: 708–711.
15. Gaskins CT, Anderson D. Statistical properties of ratios. i. empirical results. *Syst Zool* 1976; **25**: 137–148.
16. Jackson DA, Harvey HH, Somers KM. Ratios in aquatic sciences: Statistical shortcomings with mean depth and the morphoedaphic index. *Can J Fish Aquat Sci* 1990; **47**.
17. Olsson PA, Bååth E, Jakobsen I. Phosphorus effects on the mycelium and storage structures of an arbuscular mycorrhizal fungus as studied in the soil and roots by analysis of fatty acid signatures. *Appl Environ Microbiol* 1997; **63**: 3531–3538.
18. Van Aarle IM, Olsson PA. Fungal Lipid Accumulation and Development of Mycelial Structures by Two Arbuscular Mycorrhizal Fungi. *Appl Environ Microbiol* 2003; **69**: 6762–6767.
19. Oksanen J, Guillaume Blanchet F, Friendly M, Kindt R, Legendre P, McGlinn D, et al. Vegan: Community ecology package. R version 2.5-6. 2019.
20. Manzoni S, Ding Y, Warren C, Banfield CC, Dippold MA, Mason-Jones K. Intracellular storage reduces stoichiometric imbalances in soil microbial biomass – a theoretical exploration. *Front Ecol Evol* 2021; **9**: 1–21.
